# Acceptability of intrapartum ultrasound by mothers in an African population

**DOI:** 10.1007/s40477-019-00382-5

**Published:** 2019-05-08

**Authors:** Yaw Amo Wiafe, Bill Whitehead, Heather Venables, Edward T. Dassah

**Affiliations:** 1 College of Health and Social Care, University of Derby, Derby, UK; 2 Department of Medical Diagnostics, Kwame Nkrumah University of Science and Technology, Derby, Ghana; 3 Department of Obstetrics and Gynecology, Komfo Anokye Teaching Hospital, Kumasi, Ghana; 4 School of Public Health, Kwame Nkrumah University of Science and Technology, Kumasi, Ghana

**Keywords:** Transperineal ultrasound, Digital vaginal examination, Labour, Evidence-based practice, Ghana

## Abstract

**Introduction:**

Intrapartum ultrasound is gaining high acceptance by many women as another method for assessing labour progression. Despite growing evidence of the effectiveness of ultrasound in labour, the acceptance of intrapartum ultrasound has not been previously investigated in black Africans.

**Aim:**

This study aimed to determine women’s acceptance of intrapartum ultrasound and their preference for transperineal ultrasound or digital vaginal examination (digital VE) in Ghana.

**Methods:**

An analytical cross-sectional study was conducted among mothers who had had both digital VE and transperineal ultrasound during labour in a tertiary hospital. Information about their sociodemographic characteristics, experience with, and preference for ultrasound or digital VE in labour using a pretested structured questionnaire was obtained. Their experiences were categorised as ‘tolerable, ‘quite uncomfortable’ or ‘very uncomfortable’. Categorical variables were compared using Fisher’s exact test. A *p* value < 0.05 was considered statistically significant.

**Results:**

Altogether, 196 women were recruited into the study. The mean age of the women was 26.7 years (standard deviation, 4.6 years). Nearly half (47%) of the women had never delivered before. Significantly more women considered transperineal ultrasound to be more tolerable than digital VE (66% vs. 40%; *p* < 0.001). Almost all the women (97.5%) described their experience with transperineal ultrasound to be better than digital VE, and would choose transperineal ultrasound over digital VE in the future (98.5% vs. 1.5%; *p* < 0.001).

**Discussion:**

The findings of this study are comparable to those of other related studies reported recently. This research confirms high acceptance of ultrasound in labour by mothers from different countries and across continents, implying that cultural differences do not influence women’s responses to and interest in intrapartum ultrasound.

**Conclusion:**

Most women found ultrasound in labour to be more tolerable than digital VE. Whenever possible, transperineal ultrasound should be provided as an alternative to digital VE during labour.

## Introduction

Digital vaginal examination (digital VE) is a common procedure many women undergo during labour. It is used for assessing the progress of labour, which helps in identifying those women who need special care. It became the preferred choice over rectal examination (RE) some decades ago, after research found that most women were more comfortable with it during labour [1]. However, there is still a high percentage of women who find digital VE very uncomfortable [2–5], and who would, therefore, prefer another option. In keeping with the principles of evidence-based practice, healthcare providers are encouraged to consider preferences of individual patients [6]. It is, therefore, imperative to investigate other potential alternatives to digital VE, in recognition of patient value and preference.

Ultrasonography has been proposed as a potential alternative for assessing the progress of labour [7]. A comprehensive guide to using ultrasound in labour has been published by the International Society for Ultrasound in Obstetrics and Gynecology (ISOUG) [8]. For the first time, ultrasound in labour has also been reported as highly comparable to digital VE in the measurement of cervical dilatation, fetal head station and engagement in a black African population [9, 10].

However, the acceptance of intrapartum ultrasound by black African mothers has not been previously reported. Various studies conducted on the use of digital VE suggest that some mothers dislike the examination, with cultural and religious concerns being given as the basis of their dislike [11]. Given the uniqueness of the African community in terms of cultural and social life, the African women’s views regarding ultrasound in labour are important. Research on African mothers’ acceptance of intrapartum ultrasound is needed. Therefore, this study aimed to investigate mothers’ acceptance of intrapartum ultrasound in a black African population, as well as their preference for intrapartum ultrasound or digital VE.

## Methods

This analytical cross-sectional study was conducted between April and September 2016 in the labour and delivery ward of Komfo Anokye Teaching Hospital, Ghana. The study involved postpartum women who had undergone both ultrasound and digital VE during the index delivery in order for the progress of labour to be assessed. Detailed descriptions of the ultrasound and digital VE have been provided elsewhere [10]. In brief, vaginal examination was performed by an experienced clinician and in accordance with the standard protocol for the procedure in the facility. Participants were examined with ultrasound immediately after the vaginal examination.

Ultrasound examinations were performed by an independent, experienced sonographer using a Siemens-Acuson P 300 ultrasound system. First, a transabdominal approach was used to assess the fetal head position; this involved placing a covered 2–5 MHz curvilinear transducer in transverse probe orientation at the suprapubic region of the maternal abdomen. A transperineal ultrasound was performed immediately after the transabdominal scan by asking the mother to flex her legs with knees apart. Transperineal ultrasound examination was performed using the same transducer to determine cervical dilatation and other parameters associated with the determination of head station [9, 10].

After delivery, recovered participants who had consented to intrapartum ultrasound were asked again if they were willing to complete a questionnaire regarding their view about having ultrasound versus digital VE in labour. None declined. The survey questionnaire was comprised of multiple-choice questions with independent outcome variables. Responses to questions on client experience with digital VE or transperineal ultrasound were categorised as ‘tolerable’, ‘quite uncomfortable’ and ‘very uncomfortable’. The closed-ended survey questionnaire was completed by all mothers on the day of or the day following delivery.

The study was approved by the Committee on Human Research, Publications and Ethics of the Kwame Nkrumah University of Science and Technology and Komfo Anokye Teaching Hospital. Written, informed consent was obtained from all study participants.

### Statistical analysis

Data was double-entered into a Microsoft Excel spreadsheet and exported to Stata version 14.1 (StataCorp, Texas, USA) for statistical analysis. Descriptive statistical analyses were performed and the results summarised in proportions, tables and bar charts. Categorical variables and proportions were compared using Fisher’s exact test and the test of proportions, respectively. A *p* value < 0.05 was considered to be statistically significant.

## Results

Altogether, 196 completed questionnaires were analysed. Participants ranged from 18 to 39 years, with a mean of 26.7 years (standard deviation 4.6 years). Most of the mothers had completed basic education, and about one-quarter and one-fifth had completed secondary and tertiary education, respectively. All participants had undergone at least one ultrasound scan during the antenatal period. The majority of participants (83%) were Christians, and the rest were Muslims. Most (63%) of the participants were Akan, followed by various ethnic groups from the northern part of Ghana (29%), and less than 10% were Ewes/Gas (ethnic groups predominantly in southern Ghana). This was the first delivery for almost half (48%) of the mothers, and more than 80% had experienced two or more ultrasound scans during their antenatal care. Only 1% had epidural analgesia during labour. After the assessments were done, 25 women (13%) subsequently had caesarean sections for various indications, while the remaining 171 (87%) delivered vaginally.

Nearly two-thirds (66%) of the women described their experience with having transperineal ultrasound as ‘tolerable’, almost a third (31%) thought it was ‘quite uncomfortable’, and only 3% thought it was ‘very uncomfortable’.

For their experience with digital VE, 40% of mothers thought it was tolerable and 45% thought it was ‘quite uncomfortable’, whilst 15% considered it ‘very uncomfortable’. Significantly, more mothers considered ultrasound to be more tolerable than digital VE (66% vs. 40%; *p* < 0.001).

Comparing their experiences with transperineal ultrasound and digital VE, about 97.5% of mothers indicated that ultrasound was a better experience for them than digital VE. Two mothers (1%) indicated that there was no difference between the two, whilst three (1.5%) indicated that ultrasound was worse than digital VE.

When mothers were asked whether their first choice for future intrapartum care would be ultrasound or digital VE, almost all the women indicated they would like to have ultrasound in future, and would choose ultrasound ahead of digital VE (98.5% vs. 1.5%; *p* < 0.001). The vast majority (99%) would choose to have ultrasound many times rather than digital VE. Two mothers were the exception.

The relationships between the women’s sociodemographic and reproductive health characteristics and their experience with and preference for ultrasound (compared to digital VE) are shown in Table 1.

**Table 1:** Sociodemographic characteristics and experience and preference for ultrasound compared to digital vaginal examination (VE)

Variables	Experience with ultrasound better than digital VE		Prefers ultrasound as first choice		Prefers to have ultrasound many times	
	*n* (%)	*p* value	*n* (%)	*p* value	*n* (%)	*p* value
Age group (years)		1		1		0.55
18–19	10 (100)		10 (100)		10 (100)	
20–29	127 (97.0)		129 (98.5)		130 (99.2)	
30–39	54 (98.2)		54 (98.2)		54 (98.2)	
Ethnic group		0.27		0.26		0.11
Akan	122 (98.4)		123 (99.2)		124 (100)	
Mole–Dagbani	23 (95.8)		23 (95.8)		23 (95.8)	
Hausa	17 (100)		17 (100)		17 (100)	
Grusi	15 (93.8)		15 (93.8)		15 (93.8)	
Ewe/Ga	14 (93.3)		15 (100)		15 (100)	
Education		0.04		0.03		0.11
Basic	103 (99.0)		104 (100)		104 (100)	
Secondary	49 (92.5)		50 (94.3)		51 (96.2)	
Tertiary	39 (100)		39 (100)		39 (100)	
Religion		1		0.44		0.32
Christianity	158 (97.5)		160 (98.8)		161 (99.4)	
Islam	33 (97.1)		33 (97.1)		33 (97.1)	
Parity		0.85		1		1
Nulliparous	91 (97.9)		91 (97.9)		92 (98.9)	
Primiparous	43 (97.7)		44 (100)		44 (100)	
Multiparous	57 (96.6)		57 (96.6)		58 (98.3)	
Epidural		1		1		1
Yes	2 (100)		2 (100)		2 (100)	
No	189 (97.4)		191 (98.5)		192 (99.0)	

## Discussion

This study investigated black African mothers’ acceptance of intrapartum ultrasound, as well as their preference for transperineal ultrasound or digital VE.

A significant portion of mothers described transperineal ultrasound as more tolerable than digital VE, suggesting that the majority of the women were more comfortable having the former. Therefore, it was not surprising that almost all the women reported ultrasound was a better experience for them than digital VE, and that they were willing to choose ultrasound over digital VE in future labours.

Their acceptance of transperineal ultrasound was almost universal, and not influenced by their age, religion, ethnicity or parity. Again, there was over 90% acceptance at all levels of educational attainment. Almost twice (60% vs. 35%) as many women considered digital VE to be uncomfortable compared to ultrasound. This was the basis for rejecting rectal examination in the past [1]. While most women chose digital VE over rectal examination in the past [1], women today would choose ultrasound over digital VE for the same reasons.

It is also worth noting that in spite of the 40% tolerability rate reported by respondents for digital VE, the majority still preferred ultrasound. This suggests that not only did the majority regard ultrasound as being more tolerable than digital VE, but some also preferred ultrasound for other reasons not explored by the present study. However, previous studies reported that some women complained that digital VE had become too ritualistic and intimidating for them [2, 12].

The findings of this study are comparable to those of other recent and related studies. In one Chinese study [13], intrapartum ultrasound was better tolerated by Chinese women than digital VE. Usman et al. [14] also reported better tolerance for ultrasound than digital VE in the United Kingdom. Their study involved European women (53%), Asian women (34%) and Afro-Caribbean women (11%) [14]. A study conducted by Iliescu et al. [15] in Romania had an ultrasound-in-labour acceptance rate of 97.4%, which is comparable to the 98.5% obtained by the present study. The high ultrasound acceptance rate in this study also agrees with the Turkish study by Seval et al. [16], who found that women undergoing transperineal ultrasound had significantly reduced perception of pain compared to those undergoing digital VE. These studies confirm that there is a high acceptance of ultrasound in labour by mothers in different countries and across continents, implying that cultural differences do not appear to influence women’s responses.

Again, while in the study by Iliescu et al. [15], over 50% of the women were given epidural analgesia, the acceptance rate for ultrasound was almost the same as the present study, which had only 1% on epidural analgesia. This suggests that the acceptance rate for ultrasound in labour is not significantly influenced by the provision of adequate analgesia during labour.

This presents caregivers with the challenge of making ultrasound readily available as an alternative to digital VE during labour. Ultrasonography is a widely used diagnostic imaging modality in many clinical disciplines. It is already the imaging modality of choice for many obstetric conditions that have traditionally been diagnosed by physical (clinical) examination. However, its use in labour is still not widespread. A number of recent studies have shown that ultrasound is more accurate and reproducible than digital VE in labour [8]. The use of ultrasound also allows for the objective and accurate measurement of key intrapartum parameters that are used for monitoring the progress of labour [8].

To address the individual patient value and preference in this case, adequate provision has to be made for the accessibility of intrapartum ultrasonography to these women, in keeping with the principles of evidence-based practice. Evidence-based practice is ‘the conscientious, explicit, and judicious use of current best evidence in making decisions about the care of individual patients’ [6]. As evidence suggests high ultrasound accuracy and reproducibility, the high acceptance rate by women in labour now calls for high accessibility. This will ensure that the intrapartum management decisions of clinicians are based on current best evidence. The next debate is whether ultrasound can become widely available to women, especially in low-resource settings.

The main limitation of this study was that some mothers had more than one digital VE before delivery, while only one transperineal ultrasound was given to each participant. Having more than one digital VE may have influenced their experience with digital VE relative to a single ultrasound examination. A design that ensures a comparable number of ultrasound and vaginal examinations would better reflect women’s experiences of both modalities. In spite of this limitation, the results of our study were comparable to those of the study by Iliescu et al. [15], in which the women had the same number of ultrasounds as digital VE.

In conclusion, most mothers in this African population accept the use of ultrasound in labour. Therefore, in addressing the individual patient value and preferences, ultrasound in labour should be available as an alternative method for assessing the progress of labour, whenever possible. Challenges associated with the introduction of transperineal ultrasound in labour, especially in low-resource settings, need to be investigated.
